# Towards monitoring dysplastic progression in the oral cavity using a hybrid fiber-bundle imaging and spectroscopy probe

**DOI:** 10.1038/srep26734

**Published:** 2016-05-25

**Authors:** Gage J. Greening, Haley M. James, Mary K. Dierks, Nontapoth Vongkittiargorn, Samantha M. Osterholm, Narasimhan Rajaram, Timothy J. Muldoon

**Affiliations:** 1 Department of Biomedical Engineering, University of Arkansas, Fayetteville, Arkansas 72701, USA; 2 Department of Chemistry and Biochemistry, University of Arkansas, Fayetteville, Arkansas 72701, USA; 3 Department of Biological Sciences, University of Arkansas, Fayetteville, Arkansas 72701, USA

## Abstract

Intraepithelial dysplasia of the oral mucosa typically originates in the proliferative cell layer at the basement membrane and extends to the upper epithelial layers as the disease progresses. Detection of malignancies typically occurs upon visual inspection by non-specialists at a late-stage. In this manuscript, we validate a quantitative hybrid imaging and spectroscopy microendoscope to monitor dysplastic progression within the oral cavity microenvironment in a phantom and pre-clinical study. We use an empirical model to quantify optical properties and sampling depth from sub-diffuse reflectance spectra (450–750 nm) at two source-detector separations (374 and 730 μm). Average errors in recovering reduced scattering (5–26 cm^−1^) and absorption coefficients (0–10 cm^−1^) in hemoglobin-based phantoms were approximately 2% and 6%, respectively. Next, a 300 μm-thick phantom tumor model was used to validate the probe’s ability to monitor progression of a proliferating optical heterogeneity. Finally, the technique was demonstrated on 13 healthy volunteers and volume-averaged optical coefficients, scattering exponent, hemoglobin concentration, oxygen saturation, and sampling depth are presented alongside a high-resolution microendoscopy image of oral mucosa from one volunteer. This multimodal microendoscopy approach encompasses both structural and spectroscopic reporters of perfusion within the tissue microenvironment and can potentially be used to monitor tumor response to therapy.

Intraepithelial dysplastic progression within the oral mucosa is a dynamic process that typically arises at the basement membrane and is classified into stages based on how far it has spread towards the upper epithelial layers.^1^,^2^,^3^,^4^ For example, mild dysplasia occurs in the basal epithelial layers, directly above the basement membrane. As dysplasia progresses upwards towards the apical epithelial surface, the stages are characterized as moderate and severe (or carcinoma *in-situ*), respectively^2^,^3^,^4^. These stages are not considered invasive cancer since they have not yet penetrated the basement membrane and metastasized, although the severity of dysplasia increases this risk^2^,^4^. It has been found that <5%, 3–15%, and >15% of patients with mild, moderate, and severe dysplasia, respectively, progressed to carcinoma^2^,^4^. Oral squamous cell carcinoma (OSCC) is the most common form of this carcinoma in the oral cavity and patients diagnosed with OSCC have a 5-year survival rate of less than 60–70% and this number decreases in developing countries^2^,^5^,^6^,^7^. This is because primary detection of dysplastic malignancies typically occurs upon visual inspection by non-specialized dentists, who then refer patients to specialists^5^,^8^,^9^. Diagnoses at this point are often late-stage^8^. Therefore, detection of oral dysplasia at its various stages via affordable, available, and non-invasive techniques is crucial in limiting the number of cases that progress to OSCC. Several recent non-invasive translational endoscopy-based techniques have aimed at improving detection.

One such technique is high-resolution microendoscopy, which can provide clinicians rapid, high-resolution visualization of tissue architecture and histology when compared to that of the naked eye alone. These techniques provide a step towards point-of-care “optical biopsy,” potentially reducing the number of biopsies performed each year^7^,^10^. Preclinical and clinical studies using high-resolution microendoscopy techniques have been demonstrated in various body organs including the oral cavity^7^,^11^, esophagus^12^,^13^,^14^,^15^, lower gastrointestinal tract^16^,^17^,^18^,^19^,^20^,^21^, cervix^22^,^23^, ear^24^,^25^,^26^, and liver and pancreas^27^. Furthermore, several studies have developed high-resolution imaging techniques compatible with the biopsy port of conventional white-light endoscopes, making it more attractive for clinicians to adopt these new techniques^10^,^13^,^19^. Work has also been performed in quantifying high-resolution microendoscopy image data, but for the most part this remains a qualitative screening technique^14^,^15^,^21^,^28^. The advantages of high-resolution microendoscopy are low cost, portability, and instantaneous imaging of tissue architecture. However, a drawback of high-resolution microendoscopy is lack of depth sectioning, meaning it can only resolve tissue architecture at the apical epithelial surface. More complex instrumentation does exist to overcome this drawback, including laser scanning confocal systems, but this instrumentation requires galvanometers or microelectromechanical (MEMS)-based technology to do so. Additionally, information gathered by these more complex depth-sensitive technologies are primarily qualitative^29^,^30^,^31^,^32^. High-resolution microendoscopy can thus benefit from additional depth sensitive modalities since mild and moderate dysplasia are often sub-epithelial surface phenomena, but relatively simple and quantitative techniques are desirable.

One depth sensitive technique that has demonstrated diagnostic efficacy is diffuse reflectance spectroscopy (DRS), a well-established method capable of non-invasively quantifying volume-averaged tissue optical parameters using simple probe designs^33^,^34^,^35^,^36^,^37^,^38^,^39^. Raw DRS data is given in terms of reflectance, that is, the percentage of light recovered from a detection fiber to light delivered by a source fiber. Studies have shown that volume-averaged optical properties, such as reduced scattering coefficient (μ_s_′) and absorption coefficient (μ_a_) can be determined from *in vivo* samples^34^,^38^,^40^,^41^,^42^,^43^,^44^,^45^,^46^,^47^. It should be noted that these extracted values are based on the delivery and collection of light throughout an often inhomogeneous layered media, such as tissue, and extracted optical properties thus represent volume averaged, rather than axially resolved, values. Several *in vivo* DRS studies have extracted other clinically relevant optical parameters including blood volume fraction, hemoglobin concentration, oxygen saturation, mean blood vessel diameter, nicotinamide adenine dinucleotide (NADH) concentration, and tissue thickness^34^,^35^,^36^,^37^,^48^,^49^,^50^,^51^,^52^. Furthermore, DRS is an appealing non-invasive screening technique because it is sensitive to optical changes beneath the apical tissue layer^33^,^34^,^35^,^36^,^37^,^38^,^39^,^40^,^41^,^42^,^43^,^44^,^45^,^46^,^47^,^48^,^49^,^50^,^51^,^52^. However, a drawback of DRS is inability to spatially resolve tissue architecture.

We have recently reported on a probe-based technique that combines high-resolution microendoscopy imaging, and a form of DRS called broadband sub-diffuse reflectance spectroscopy (sDRS) within a single fiber-bundle^29^,^53^. The term “sub-diffuse reflectance” is used here to be distinguished from “diffuse reflectance” to describe the cases in which our source-detector separations (SDS) are less than one reduced mean-free path within a sample, which will vary based on a sample’s optical properties^40^,^54^,^55^,^56^,^57^,^58^. This hybrid fiber-bundle spectroscopy and imaging probe is capable of co-registering qualitative high-resolution images of tissue surface microarchitecture with complimentary quantitative and depth-sensitive spectral data^29^,^53^. Furthermore, our design uses two SDSs (shallow and deep channels) to collect data at two different sampling depths with the goal of sampling different tissue volumes. Therefore, the high-resolution imaging modality may be beneficial in providing image data of later stage moderate and severe dysplasia while the sDRS modality may be sensitive to tissue optical changes associated with early dysplasia arising at the basement membrane^29^.

In this manuscript, we validate the sDRS portion of the quantitative hybrid imaging and spectroscopy microendoscope and present a pilot phantom and pre-clinical study to extract *in vivo* optical parameters of the human oral mucosa. First, a set of calibration phantoms was used to generate reflectance lookup tables (LUT) describing the relationship between reflectance and optical properties (μ_s_′ and μ_a_) for the sDRS modality^40^. Then, to validate the LUT, the probe and LUT-based inverse model was used to extract μ_s_′ and μ_a_ from a set of hemoglobin-based validation phantoms with known μ_s_′ and μ_a_^40^. Extracted optical properties were compared to theoretical values and reported as percent errors. Next, we quantify sampling depth for the shallow and deep SDSs of the sDRS modality and validate results using the same calibration and validation phantoms^59^. Following this, we present a simple phantom study simulating the physical layered progression from healthy tissue to severe dysplasia to show how reflectance changes with an optically scattering heterogeneity buried at various depths^1^,^2^,^4^. Finally, the LUT-based inverse model was demonstrated on *in vivo* human oral mucosa from thirteen healthy volunteers in a laboratory setting to determine volume-averaged scattering exponent, hemoglobin concentration, oxygen saturation, and sampling depth. The extracted *in vivo* quantitative optical parameters were compared to an *in vivo* high-resolution image of healthy, non-keratinized oral tissue. These studies validate our hybrid fiber-bundle imaging and spectroscopy technique and demonstrate the translational potential to a clinical setting. This technique can potentially be used to for diagnostic purposes as well as dynamically monitoring personalized tumor response to therapy.

## Materials and Methods

### Instrumentation

**Figure 1:**
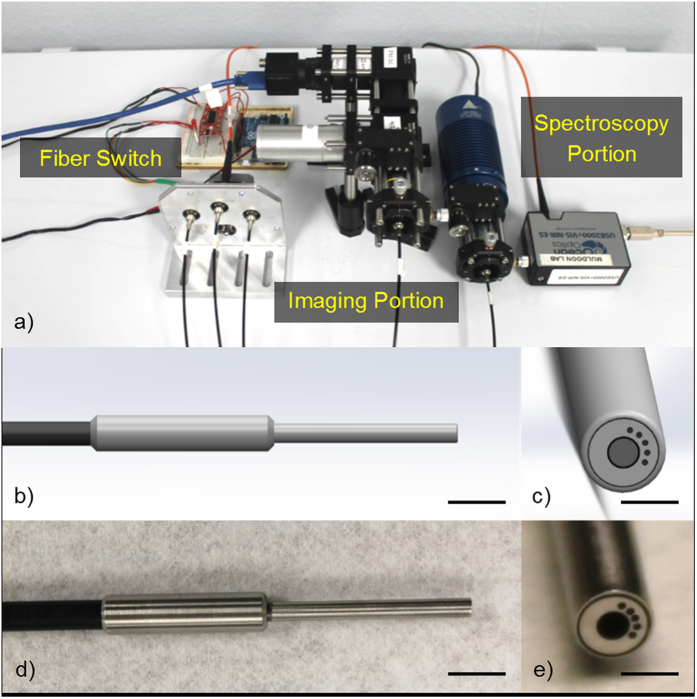
Representation of the hybrid fiber-bundle imaging and spectroscopy system showing (**a**) the major instrumentation components including (from left to right) fiber switch, imaging portion, and spectroscopy portion, (**b**) a SolidWorks representation of the distal probe (scale bar = 1 cm) showing the (**c**) en face view of the central 1 mm-diameter image fiber and 5 surrounding 200 μm multimode fibers (scale bar = 2.5 mm), (**d**) distal probe (scale bar = 1 cm), and (**e**) en face view of the distal probe tip (scale bar = 2.5 mm).

The first objective of this study was to design the multimodal instrumentation and associated contact fiber-bundle probe to co-register qualitative image data with quantitative spectroscopy data^29^,^53^. For the high-resolution fluorescence imaging modality, a 455 nm LED (20 FWHM) light source (Philips, USA) is coupled through a 1 mm-diameter image fiber (FIGH-50-1100N, Myriad Fiber Imaging, USA) consisting of approximately 50,000 individual 4.5 μm-diameter fibers. The distal blue 455 nm LED light excites a contrast agent, such as proflavine, which emits fluorescence signal (peak emission of ~515 nm with quantum efficiency of ~0.5) back into the image fiber and is delivered to an 8-bit monochrome CMOS camera (FL3-U3-32S2M-CS, Point Grey, Canada)^29^,^53^,^60^. A filter set (Chroma Technology Corp., USA) separates the excitation and emission signals. Next, for the sub-diffuse reflectance spectroscopy (sDRS) modality, a tungsten-halogen lamp (HL-2000-LL, Ocean Optics, USA) delivers broadband light through a multimode 200 μm (NA = 0.22) delivery fiber (FBP200220240, Molex Inc., USA) to a material. Four adjacent and identical 200 μm (NA = 0.22) multimode fibers, with center-to-center source-detector separations (SDS) of 374, 730, 1,051, and 1,323 μm, respectively, collect the broadband sub-diffusely reflected light and deliver it to a spectrometer (USB2000+UV-VIS, Ocean Optics, USA) with a spectral resolution of 0.35 nm^29^. Although this technique is capable of having four SDSs, only two (374 and 730 μm) are presented in the following studies. Figure 1 shows the instrumentation and probe design.

### Generation of and validation of lookup tables for volume-averaged optical property extraction

The second objective of this study was to use the sDRS modality to extract volume-averaged optical parameters. To accomplish this, reflectance lookup tables (LUTs) were generated describing the relationship between absolute reflectance and optical properties (μ_s_′ and μ_a_) for the two SDSs (374 and 730 μm). The target ranges of the LUTs were μ_s_′ and μ_a_ between 5–26 cm^−1^ and 0–10 cm^−1^, respectively. These LUTs required calibration phantoms of similar order of magnitude as biological tissue^40^,^61^.

Calibration phantoms were constructed to exceed the target range using deionized water as the solvent^40^. The scattering agent was 1.0 μm-diameter polystyrene microspheres (07310-15, Polysciences, USA) and the associated μ_s_′ range (3–31 cm^−1^) was calculated using Mie theory^49^,^50^,^62^. The absorbing agent was a combination of yellow, red, and blue food dye (McCormick & Company, USA), in ratio of 20:6:2, which contained propylene glycol, Yellow 5, Red 40, Red 3, Blue 1, and 0.1% propylparaben. The μ_a_ range (0–47 cm^−1^) was calculated by measuring the dye solution in deionized water using a spectrophotometer (5102-00, PerkinElmer, USA) and Beer’s Law. All calibration phantoms were homogenous so μ_s_′ and μ_a_ were identical throughout the phantom volume.

A total of 12 liquid calibration phantoms was created which was sufficient to build the LUTs. Six of the 12 phantoms were considered “scattering-only” and contained only deionized water and polystyrene microspheres without dye. Deionized water and polystyrene microspheres were gently mixed inside 7 mL scintillation vials (66022-300, VWR, USA) to yield six μ_s_′ ranges of 3.0–4.9, 4.4–7.1, 6.4–10.2, 9.2–14.7, 13.2–21.2, and 19.5–31.0 cm^−1^. These values were chosen so there was sufficient overlap between the maximum μ_s_′ value of one phantom at 450 nm and the minimum μ_s_′ value of another phantom at 750 nm. Sufficient overlap was determined such that the minimum μ_s_′ value of one phantom was no greater than 90% of the maximum μ_s_′ value of the proceeding phantom. This ensured the six scattering-only phantoms spanned a continuous μ_s_′ range. The following equation expresses this relationship in which *n* is the phantom number.


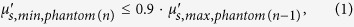


The remaining six phantoms contained both polystyrene microspheres and the dye combination. Deionized water, polystyrene microspheres, and dye were gently mixed inside 7 mL scintillation vials to yield a continuous μ_s_′ range of 3–31 cm^−1^ and continuous μ_a_ range of 0–47 cm^−1^. The wavelength-dependent variations in μ_s_′ and μ_a_ provide the wide range of scattering and absorbing values.

To generate the reflectance LUTs, the probe was placed in each phantom so it was completely submerged at a distance of 2 cm from the bottom of the 7 mL scintillation vial. Broadband sDRS data (450–750 nm) were recorded at each SDS (374 and 730 μm) with an integration time of 400 ms. Five spectra were averaged for all measurements. Spectra were converted to absolute reflectance values by calibrating with a Spectralon^®^ 20% diffuse reflectance standard (SRS-20-010, Labsphere, USA) which was spectrally flat between 200–2600 nm. All spectra were corrected for background noise^33^,^34^,^40^,^47^,^49^. After acquiring absolute reflectance spectra at a resolution of 0.35 nm, the LUTs relating reflectance (R) to μ_s_′ and μ_a_ were generated using MATLAB. Raw data from the 12 calibration phantoms (C.P. #1-12) was interpolated to generate a color-mapped mesh with an optical property resolution of 0.02 cm^−1^. The reflectance LUTs were interpolated in the target μ_s_′ and μ_a_ ranges of 5–26 cm^−1^ and 0–10 cm^–1^, respectively.

To validate the reflectance LUTs, a set of liquid validation phantoms with known optical properties was built of similar order of magnitude as biological tissue^40^,^61^. Validation phantoms were constructed in a similar manner to calibration phantoms, but contained bovine hemoglobin (H2625, Sigma-Aldrich, USA), rather than food dye, as the absorbing agent. The μ_s_′ was calculated using Mie theory and μ_a_ was calculated by measuring a solution of bovine hemoglobin in deionized water using a spectrophotometer (5102-00, PerkinElmer, USA) and Beer’s Law. It was necessary to validate the LUTs using a different absorber and different scattering ranges than those used to generate the LUTs so that the interpolated range of the LUTs were tested. All validation phantoms were homogenous so μ_s_′ and μ_a_ were identical throughout the phantom volume.

**Figure 2:**
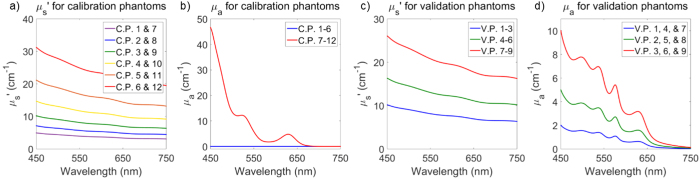
Comparison of the optical properties of the (**a,b**) 6 × 2 (12 total) calibration phantoms (C.P.) and the (**c,d**) 3 × 3 (9 total) validation phantoms (V.P.). Calibration phantoms were made with polystyrene microspheres and a combination of yellow, red, and blue dye and the validation phantoms were made with polystyrene microspheres and bovine hemoglobin as the scattering and absorbing agents, respectively. Calibration phantoms had μ_s_′ spanning 3–31 cm^−1^ and μ_a_ spanning 0–47 cm^−1^ and the validation phantoms had a μ_s_′ spanning 5–26 cm^−1^ and μ_a_ spanning 0–10 cm^−1^ to validate the target LUT range.

A 3 × 3 (9 total) set of validation phantoms was created, corresponding to three μ_s_′ ranges and three μ_a_ ranges. Deionized water, polystyrene microspheres and diluted bovine hemoglobin were gently mixed inside 7 mL scintillation vials. This yielded μ_s_′ values from 5–26 cm^−1^ and μ_a_ values from 0–10 cm^–1^ to validate 100% of the reflectance LUTs. Figure 2 shows the μ_s_′ and μ_a_ for the calibration phantoms (C.P. 1-12) and validation phantoms (V.P. 1–9).

Broadband sDRS data on validation phantoms were collected in the same method as the calibration phantoms. The LUT-based inverse model was used to extract μ_s_′ and μ_a_ from the validation phantoms. Theoretical optical properties of the validation phantoms were compared to extracted optical properties and reported as percent errors. To quantify percent errors, the LUT-based inverse model extracted μ_s_′ and μ_a_ for the 3 × 3 validation phantoms at a spectral resolution of 0.35 nm and percent errors were calculated using the following formulas,


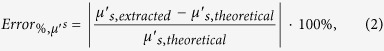



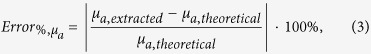


### Generation of and validation of lookup tables for sampling depth quantification

The third objective of this study was to determine the sampling depth of the sDRS modality. To accomplish this, sampling depth lookup tables (LUTs) were generated describing the relationship between sampling depth and volume-averaged optical properties (μ_s_′ and μ_a_) for the two SDSs (374 and 730 μm). The target ranges of the sampling depth LUTs were μ_s_′ and μ_a_ between 5–26 cm^−1^ and 0–10 cm^−1^, respectively. The same calibration phantoms as described previously were used to generate the sampling depth LUTs.

A highly absorbing phantom layer (μ_a_ ≥ 100 cm^−1^ for all wavelengths between 450–750 nm) was created in a 5 mL beaker using 6.5% w/w India Ink in PDMS^59^,^63^. Contributions from specular reflection were proven negligible by placing the probe in contact with the absorbing layer and acquiring sDRS data between distances of 0–2 mm in 50 μm increments^59^.

**Figure 3:**
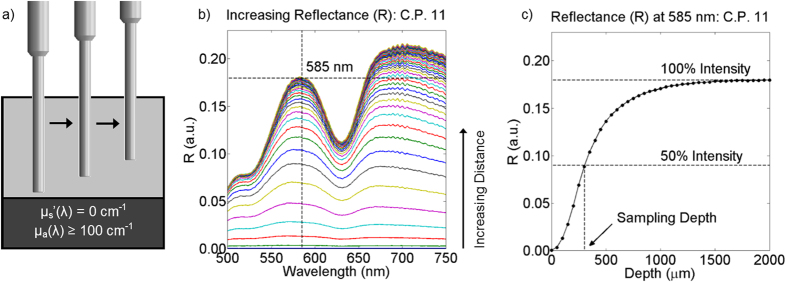
The probe is placed (**a**) in contact with the highly absorbing (μ_a_ ≥ 100 cm^−1^ for 450–750 nm) inside a 5 mL beaker and translated upwards in 50 μm increments to (**b**) acquire sDRS data from a calibration phantom (C.P. 11) at a 374 μm SDS. (**c**) Representative data from the 374 μm SDS shows the percentage of photons not reaching the highly absorbing layer as a function of depth for C.P. 11 at 585 nm. Sampling depth is defined as the depth reached by 50% of photons.

Next, the six dye-containing calibration phantoms (Fig. 2, C.P. 7–12) were placed on top of the highly absorbing layer within the beaker. Spectra (450–750 nm) at each SDS were taken by varying the distance of the probe-tip and absorbing layer between 0–2 mm in 50 μm increments^59^. Sampling depth is been defined as the depth reached by 50% of photons^59^. At a certain probe-absorbing layer distance (around 2 mm), there were no significant changes in signal intensity, meaning that nearly 100% of incident photons were not reaching the highly absorbing layer. Figure 3 shows how sampling depth was quantified for the sDRS modality in representative data^59^. As the probe is translated away from the absorbing layer, as shown in Fig. 3a, reflectance increases until plateauing as shown in Fig. 3b. A depth (x-axis) can then be identified that correlates with the 50% cutoff point (y-axis) which is defined as the sampling depth as shown in Fig. 3c 59.

The process from Fig. 3 was repeated for all wavelengths at a spectral resolution of 0.35 nm for the 6 calibration phantoms (C.P. 7–12). Raw data was interpolated in Matlab to generate a color-mapped mesh with a maximum optical property resolution of 0.02 cm^−1^. The sampling depth LUTs were interpolated in a target μ_s_′ range of 5–26 cm^−1^ and μ_a_ range of 0–10 cm^−1^.

To validate sampling depth, spectra (450–750 nm) at each SDS of the previously described validation phantoms were acquired by varying the distance of the probe-tip and absorbing layer between 0–2 mm in 50 μm increments. To quantify percent errors, sampling depths of the validation phantoms were compared to the sampling depths (D) from the calibration phantoms. Percent errors were calculated using the following formula,


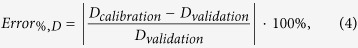


### Semi-infinite phantom model of dysplastic progression

**Figure 4:**
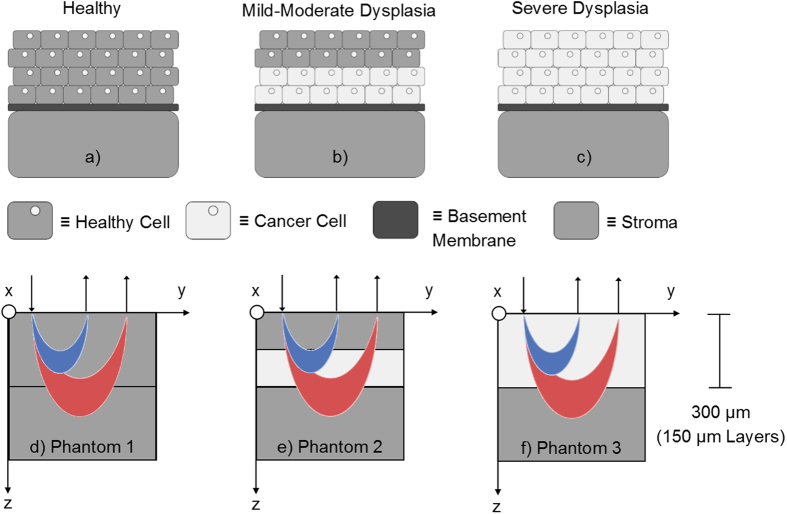
A simplified representation of dysplastic proliferation arising at the basement membrane in the oral cavity (**a–c**) showing normal cells (gray with nuclei), dysplastic cells (light gray with nuclei), basement membrane (dark gray), and the stroma (gray). The associated dysplasia-mimicking phantom models (**d–f**) simulate this progression. Two SDSs (374 and 730 μm) deliver and collect broadband light at different depths (detected photons shown here as blue and red crescents, respectively). Each of thin phantom layers was 150 μm thick for a total phantom thickness of 300 μm to simulate the thickness of oral epithelium.

Once optical property extraction and sampling depth were validated, we tested the capabilities of the sDRS modality of the hybrid fiber-bundle in a dysplasia-mimicking phantom model^1^. Figure 4a–c shows a simplified representation of dysplastic progression starting at the basement membrane and proliferating upwards into surrounding healthy tissue^2^,^3^. Early dysplasia is known to significantly increase epithelial scattering by nearly two-fold^64^,^65^,^66^. To simulate this phenomenon, three solid scattering-only phantoms, shown in Fig. 4d–f, were created^1^. Since scattering contributes much more to reflectance intensity compared to absorption, the μ_a_ was held constant at 0 cm^−1 ^66. Additionally, the phantom “epithelia” was made to be 300 μm thick to approximately simulate the thickness of oral mucosa^67^. With the understanding that the 374 and 730 μm SDSs sample different depths, it was expected that the 374 μm SDS may be more sensitive to shallower, epithelial-confined scattering changes associated with early dysplasia.

The three phantom models have a semi-infinite geometry, a common geometry used in various models of photon transport in tissues with sub-surface optical heterogeneities^1^. The semi-infinite geometry requires an optically thick base layer (bottom gray layer in Fig. 4d–f) that can be considered infinitely thick in the *z* direction since no photons penetrate through this layer. In this experiment, the semi-infinite base layer was 1 cm thick. Additionally, all layers can be considered infinite in the *x* and *y* directions since no photons penetrate laterally outside this plane^1^.

Phantoms were created using poly(dimethylsiloxane) (PDMS) as the substrate material, and titanium dioxide (TiO_2_) as the scattering agent. PDMS was used because of its optical clarity (μ_s_′ and μ_a_ = 0 cm^−1^ between 500–750 nm), comparable refractive index to human tissue (~1.4), optical stability over time, physical durability, and ability to form multilayer geometries^68^. Since μ_s_′ contributes to reflectance intensity much more than μ_a_, no absorbing agent was used^66^.

The semi-infinite layer and 150 μm thick healthy tissue-mimicking layers were designed with 0.25% w/w TiO_2_ in PDMS (2.5 mg TiO_2_ per 1.0 g PDMS) to yield a μ_s_′ of ~7 cm^−1^ at 630 nm which is comparable to healthy tissue^68^,^69^. The 150 μm thick dysplasia-mimicking layers were designed with 0.50% w/w TiO_2_ in PDMS (5.0 mg per 1.0 g PDMS) to yield a μ_s_′ of ~14 cm^−1^ at 630 nm^68^,^69^. This represented a two-fold increase in scattering which is representative of the increased scattering ratio of dysplastic to healthy epithelial tissue^64^,^65^,^66^. For each geometry in Fig. 4, two 150 μm layers were stacked to generate the desired phantom^67^,^68^. The total phantom “epithelial” thickness was thus 300 μm, not including the “stromal” semi-infinite base layer, which was 1 cm thick. All thin phantom layers were created using a previously described spin coating technique^67^,^68^.

The volume-averaged μ_s_′ was extracted between 500–750 nm for each phantom. Ten sDRS measurements were averaged for each geometry (Phantoms 1–3) and SDS with an integration time of 500 ms. We hypothesized that the 374 μm SDS would show larger deviations in volume-averaged μ_s_′ compared to the 730 μm SDS because the changes in scattering were confined to the upper 300 μm of the phantom. The 730 μm would be sampling significantly more into the underlying “stromal” semi-infinite layer, in which μ_s_′ was held constant for this experiment. Results from this study were expected to indicate that the shorter SDS would be more sensitive to scattering changes associated with dysplastic epithelia.

### *In vivo* assessment of oral structural and optical properties

The final objective of this study was to extract optical parameters from *in vivo* oral mucosa and elucidate the differences of the optical parameters for each SDS (374 and 730 μm). The multimodal technique was demonstrated in the inner lip of thirteen healthy volunteers, with no history of tobacco use, between the ages of 18–35. Institutional Review Board approval (IRB #15-09-149) was obtained from the Human Subjects Research program at the University of Arkansas for all aspects of this study. The methods described were carried out in accordance with the approved guidelines, and informed consent was obtained from all participants.

Extracting optical parameters required two steps. First, *in vivo* data acquisition was carried out with custom LabVIEW software^29^. The probe was directly placed in contact with the inner lip and broadband sDRS were acquired at both SDSs (374 and 730 μm). The tungsten-halogen lamp delivered 0.35 mW of power at the probe tip for 500 ms. Additionally, in one volunteer, a single high-resolution fluorescence image was taken using topical proflavine (0.01% w/v in saline) as a contrast agent with an exposure of 100 ms and gain of 5 dB, thus demonstrating the capability of the probe to sequentially and non-invasively extract image and optical property data. Second, for post-processing, raw broadband sDRS data was imported into custom MATLAB software which was integrated with the LUT-based inverse model and sampling depth LUT to extract optical parameters. The use of this post-processing algorithm to extract optical parameters has been previously described^34^,^35^,^36^.

The optical parameters extracted in this study were volume-averaged scattering exponent (B), hemoglobin concentration ([Hb]), and oxygen saturation (SaO_2_). Sampling depth was also quantified which is a function of the underlying optical parameters^35^,^40^,^51^,^59^. The scattering exponent relates to the size of a tissue’s scattering particles, and thus can provide reasoning for changes in scattering when comparing groups within the same SDS^70^. Hemoglobin concentration and oxygen saturation are commonly derived measurements in optical spectroscopy to assess angiogenesis, and since blood vessel density has been shown to increase as oral tissue progresses from normal to dysplastic, extracting these parameters was important^71^. These optical parameters and their relation to μ_s_′ and μ_a_ are given in equations 5 and 6. The μ_s_′ was calculated based on the following equation,


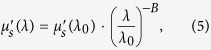


where *μ*_*s*_*’(λ)* is the reduced scattering coefficient (cm^−1^) at any wavelength, *λ* is a wavelength (nm), *λ*_*0*_ is 630 nm, and *B* is the scattering exponent^51^. The μ_a_ was calculated based on the following equation,


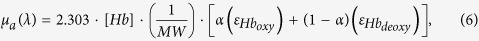


where *μ*_*a*_ is the absorption coefficient (cm^−1^) , [Hb] is the bulk tissue hemoglobin concentration (mg/mL), *MW* is the gram molecular weight of hemoglobin which is assumed to be 64,500 g/mole^72^, *α* is the bulk tissue oxygen saturation, and *ε* is the molar extinction coefficient (cm^−1^M^−1^) of oxygenated hemoglobin (Hb_oxy_) and deoxygenated hemoglobin (Hb_de-oxy_). Some groups have also included a packaging correction factor when calculating μ_a_ for sampling wavelengths below 450 nm, but this was shown to have no impact on the LUT-based inverse model fits presented here since spectra were taken between 500–750 nm^35^.

**Figure 5:**
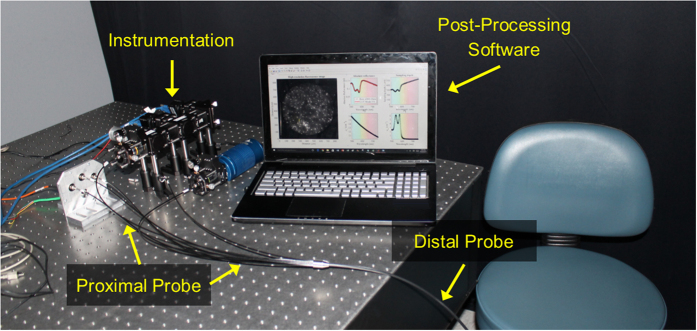
An image of the experimental setup showing the optical instrumentation, post-processing software based in MATLAB showing a high-resolution fluorescence image of the inner lip, LUT-based inverse model fit of raw reflectance data, sampling depth, μ_s_′, and μ_a_ from one volunteer (image center), and the proximal and distal hybrid fiber-bundle probe.

Figure 5 shows the experimental setup with the instrumentation, hybrid fiber-bundle probe, and post-processing software. For this experiment, it was hypothesized that the 730 μm SDS would yield reduced B values due longer SDSs having greater reflectance from longer wavelengths. Alternatively, the 730 μm SDS should yield greater [Hb] values because of increased sampling into the sub-epithelia, where the blood vessels exist^51^,^69^. SaO_2_ was expected to be comparable when sampling at different depths since changes in SaO_2_ have been shown to not be depth dependent^73^. Finally, we expected increased sampling depth for the longer SDS^51^,^59^. Results from this study were expected to show the value of including two different sub-diffuse reflectance spectroscopy SDSs along with a high-resolution fluorescence imaging capability.

## Results

### Generation of and validation of lookup tables for volume-averaged optical property extraction

**Figure 6:**
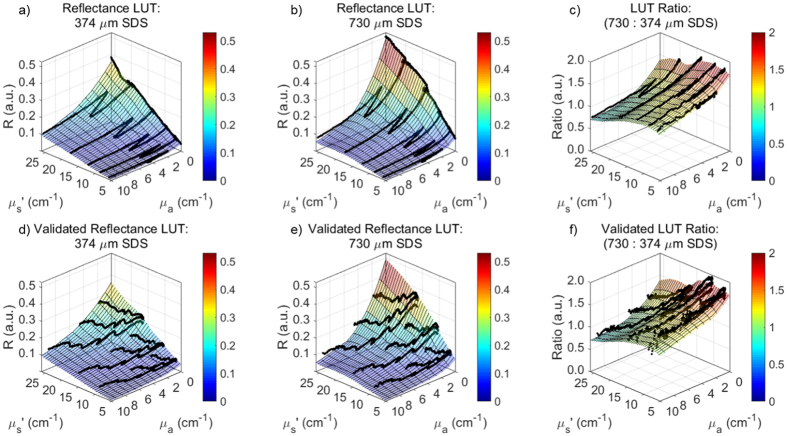
100% (μ_s_′ = 5–26 cm^−1^, μ_a_ = 0–10 cm^−1^) of both reflectance LUTs were validated with acceptable percent errors less than 10%. Following validation, optical properties can be reliably extracted from samples with unknown optical properties using the LUT-based inverse model. (**a,b**) Reflectance LUTs were interpolated with raw data from calibration phantoms and (**c**) shows a ratio of the 730 μm SDS to 374 μm SDS LUTs. (**d,e**) Reflectance LUTs were validated with raw data from the bovine hemoglobin-based validation phantoms and (**f**) shows the validated ratio of the 730 μm SDS to 374 μm SDS LUTs.

Figure 6a,b shows the reflectance LUTs (μ_s_′ = 5–26 cm^−1^ and μ_a_ = 0–10 cm^−1^) overlaid with the respective reflectance data from the dye-based calibration phantoms. Similarly, Fig. 6d,e shows the reflectance LUTs overlaid with the respective data from the bovine hemoglobin-based validation phantoms. Validation phantom data that perfectly overlays the LUT would indicate a 0% error; however, minor errors do exist. Additionally, Fig. 6c,f shows a ratio of the 730 to 374 μm SDS LUTs. The mean ratio is 1.14, with a standard deviation of 0.27, indicating a variable reflectance ratio as μ_s_′ and μ_a_ vary. Notice that at high reduced mean free paths (low μ_s_′ and μ_a_) in Fig. 6c,f, the reflectance ratio is at a maximum of 1.69, and at low reduced mean free paths (high μ_s_′ and μ_a_), the reflectance ratio is at a minimum of 0.58. This trend supports the observation that longer SDSs are more sensitive to lower scattering values, especially at longer wavelengths. Similarly, shorter SDSs are more sensitive to higher scattering values. Thus, this reflectance ratio trend supports the validity of our LUTs.

The LUT-based inverse model correctly estimated μ_s_′ of the validation phantoms with average percent errors of 1.6% and 2.5% for the 374 and 730 μm SDS, respectively. Minimum and maximum percent errors for μ_s_′ extraction were 0.1% and 5.3% for the 374 μm SDS and 1.2% and 11.4% for the 730 μm SDS, respectively. Additionally, the LUT-based inverse model correctly estimated μ_a_ of the validation phantoms with average percent errors of 4.2% and 7.2% for the 374 and 730 μm SDS, respectively. Minimum and maximum percent errors for μ_a_ extraction were 2.1% and 18.4% for the 374 μm SDS and 0.1% and 22.1% for the 730 μm SDS, respectively.

**Figure 7:**
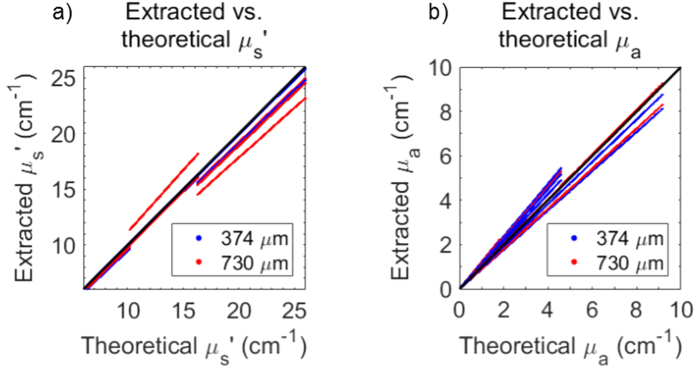
The LUT-based inverse model correctly estimated μ_s_′ with average percent errors of 1.6% and 2.5% for the 374 and 730 μm SDS, respectively, and correctly estimated μ_a_ with average percent errors of 4.2% and 7.2% for the 374 and 730 μm SDS, respectively. The ability to extract optical properties is shown with a perfect fit line.

Average percent errors were comparable to similar studies (<10%) and considered acceptable^34^,^35^,^36^,^40^,^42^,^43^,^44^,^49^,^50^,^51^. Thus, 100% of the optical property range of the LUTs were validated, and could be used to reliably extract volume-averaged optical properties from unknown samples. Figure 7 shows the ability of the reflectance LUTs to extract accurate μ_s_′ and μ_a_.

### Generation of and validation of lookup tables for sampling depth quantification

**Figure 8:**
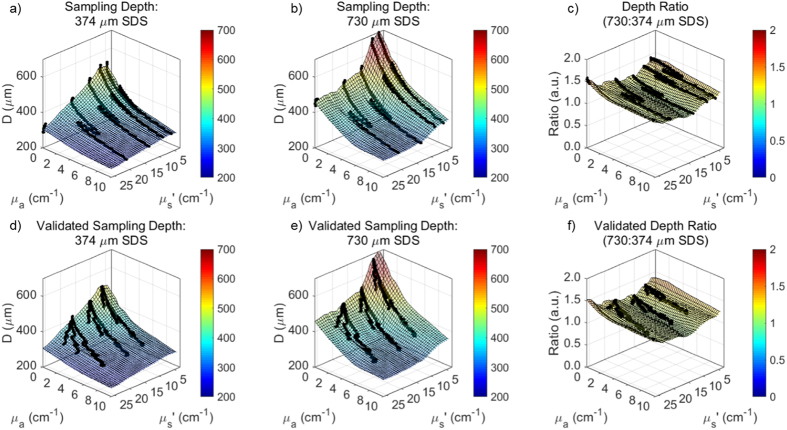
100% (μ_s_′ = 5–26 cm^−1^, μ_a_ = 0–10 cm^−1^) of both sampling depth LUTs were validated with acceptable percent errors much less than 10%. (**a,b**) Sampling depth LUTs were interpolated with raw data from calibration phantoms and (**c**) shows a ratio (1.2×) of the 730 μm SDS to 374 μm SDS sampling depths. (**d,e**) Sampling depths LUTs were validated with raw data from the bovine hemoglobin-based validation phantoms and (**f**) shows the validated ratio of the 730 μm SDS to 374 μm SDS sampling depths.

Sampling depth ranged between 240 to 530 μm and 300 to 680 μm for the 374 and 730 μm SDSs, respectively (Fig. 8). In both cases, maximum sampling depth occurred when μ_s_′ and μ_a_ were 0 cm^−1^ and minimum sampling depth occurred at the maximum μ_s_′ (26 cm^−1^) and maximum μ_a_ (10 cm^−1^) in the target range of the LUTs. After validation with hemoglobin-based validation phantoms, sampling depth was estimated with average percent errors of 1.9% and 1.6% for the 374 and 730 μm SDS, respectively. Minimum and maximum percent errors for μ_s_′ extraction were 1.8% and 5.3% for the 374 μm SDS and 1.1% and 2.1% for the 730 μm SDS, respectively. Average percent errors, all under 2%, were considered acceptable in this study. Additionally, the ratio of sampling depths for the 730 to 374 μm SDS were calculated for the entire LUT range (Fig. 8c,f). On average, the sampling depth ratio was 1.20 with a standard deviation of 0.08, and relatively flat as expected. This indicates the sampling depth of the longer SDS is approximately 1.2× that of the shorter SDS across all wavelengths.

### Extraction of sampling depth from semi-infinite phantom model of dysplastic progression

**Figure 9:**
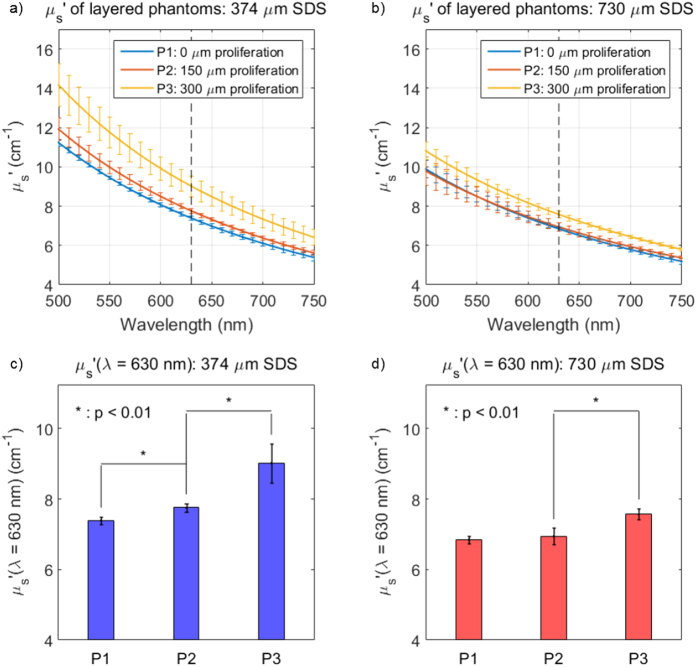
The volume-averaged μ_s_′ (**a,b**) increased as the proliferating scattering heterogeneity moved upwards towards the phantom surface (going from P1 to P3) showing a vertical line at 630 nm, in which percent increase in volume-averaged μ_s_′ was measured from. There was a significantly greater μ_s_′ increase in these values for the 374 μm SDS compared to the 730 μm SDS, indicating that the shorter SDS is more sensitive to superficial scattering changes associated with early epithelial dysplasia.

Three different phantom geometries, simulating the progression from healthy tissue to severe dysplasia, underwent sDRS evaluation using both SDSs (374 and 730 μm). Fig. 9 shows that the extracted μ_s_′ for phantom 1 (blue line) was approximately 7 cm^−1^ at 630 nm, as expected from the phantom generation protocol^68^. As the higher scattering (μ_s_′ = 14 cm^−1^) layers proliferated upwards towards the probe tip (phantoms 2 and 3), an increase in volume-averaged μ_s_′ occurred for both SDSs, although more so for the shorter SDS, as expected. For the shorter SDS, there was a significant increase in volume-averaged μ_s_′ from phantoms 1 to 2 and 2 to 3. However, for the longer SDS, there was only a significant increase in volume-averaged μ_s_′ from phantoms 2 to 3. This indicates the 374 μm SDS is more sensitive to scattering heterogeneities at upper layers compared to the 730 μm SDS.

This phenomenon is further quantified in Table 1 by the percent increase in volume-averaged μ_s_′ at 630 nm for Phantoms 1–3 for each SDS. The data indicates that the μ_s_′ percent increase for the 374 μm SDS is significantly greater compared to the 730 μm SDS. This is because the shorter SDS has a decreased sampling depth, and therefore scattering is mostly affected by more superficial heterogeneities, as seen in early dysplasia, compared to the longer SDS. However, it is important to note that the 374 μm SDS still does not exclusively sample the upper layers, as indicated by the fact that the volume-averaged μ_s_′ of phantom 3 (300 μm thick heterogeneity) is approximately 9 cm^−1^ rather than 14 cm^−1^ at the reference 630 nm. Additionally, sampling depth of the 374 μm SDS at a μ_s_′ of 14 cm^−1^ is ~400 μm, indicating a sampling depth deeper than the 300 μm scattering heterogeneity. These results demonstrate the value of including a shorter SDS for detection of more superficial scattering changes. The value of including an additional longer SDS was shown in the following section describing *in vivo* results from healthy human oral mucosa.

### *In vivo* assessment of oral structural and optical properties

**Figure 10:**
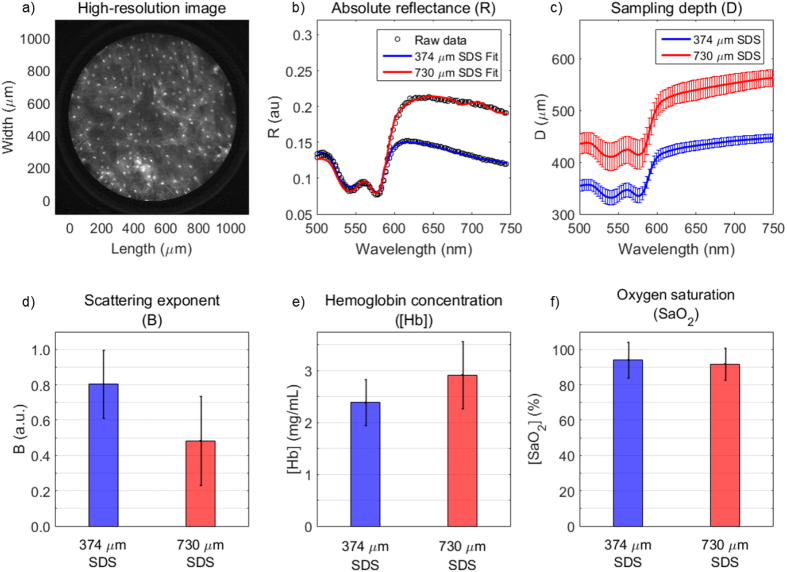
Comparison of qualitative and quantitative data acquired by the hybrid imaging and spectroscopy technique from 13 healthy volunteers showing (**a**) a high-resolution fluorescence image of apical oral mucosa from the inner lip of one volunteer (scale bar = 200 μm), (**b**) representative absolute reflectance profiles showing reflectance data and the overlaid LUT-based inverse model fits from the same volunteer from (**a**,**c**) average sampling depths for each SDS, (**d**) scattering exponent (B), (**e**) hemoglobin concentration ([Hb]), and (**f**) oxygen saturation (SaO_2_). Error bars from (**c–f**) represent standard deviation.

Thirteen volunteers underwent data collection in the oral mucosa via the hybrid imaging and spectroscopy microendoscope (Fig. 10). One high-resolution fluorescence image is presented in Fig. 10a which shows the 1 mm-diameter image circle of the image fiber in direct contact with proflavine-stained oral mucosa. Individual cell nuclei appear as distinct white spots in the image. Fig. 10b shows representative absolute reflectance data from both the 374 and 730 μm SDS from a single volunteer. Reflectance is presented as black dots and the LUT-based inverse model (Fig. 6) and an established hemoglobin absorption spectrum^72^ was used to fit the data via custom post-processing software based in MATLAB. The fitted reflectance is a function of the volume-averaged optical parameters, B, [Hb], and SaO_2_ (Eqs 5 and 6). These values are presented as averages with standard deviations from the 13 volunteers in Fig. 10d–f and Table 2. Sampling depth was quantified and presented in Fig. 10c after μ_s_′ and μ_a_ were determined using the LUT-based inverse model (Fig. 7).

The 730 μm SDS typically demonstrates increased reflectance values, especially at wavelengths greater than 600 nm, indicating a greater contribution from the red and near-infrared region at larger source-detector separations. This phenomenon was responsible for the decreased B values at the longer SDS of 0.48 compared to 0.80 of the shorter SDS as shown in Fig. 10d. Average [Hb] was significantly different at 2.39 and 2.91 mg/mL for the 374 and 730 μm SDS, respectively (Fig. 10e). These values support our hypothesis and demonstrate increased [Hb] for the longer SDS compared to the shorter SDS. Average SaO_2_ was not significantly different at 94.1% and 91.7% for the 374 and 730 μm SDS, respectively (Fig. 10f), supporting our hypothesis that oxygen saturation does not significantly vary with sampling depth. Finally, sampling depth ranged between 355 and 447 μm for the 374 μm SDS and between 435 and 563 μm for the 730 μm SDS, with the sampling depth minima occurring at the first Q-band of hemoglobin at 542 nm and the sampling depth maxima occurring at the furthest tested wavelength at 750 nm. Complete paired t-test statistics for optical parameters are shown in Table 2.

## Discussion

We have demonstrated a hybrid spectroscopy and imaging probe capable of acquiring qualitative and quantitative data by combining high-resolution microendoscopy and broadband sDRS. High-resolution fiber-bundle microendoscopy provides a highly resolved and magnified image of apical epithelial architecture in a small 1 mm-diameter field-of-view while sDRS provides quantitative optical parameters of tissue in approximately the same image region (Fig. 1). By combining these two modalities, we can co-register qualitative image data and quantitative spectral data within a single probe. Co-registration is important because this technique can be potentially used to not only detect dysplasia using two different modalities, but also to monitor personalized response of sub-surface dysplastic lesions to anti-tumor therapy at two different source-detector separations.

In this study, we designed two sets of liquid phantoms (Fig. 2) to generate and validate a LUT-based inverse model that was used to extract material optical parameters from raw sDRS data for each SDS (Fig. 6). As of the current report, the LUTs are valid for μ_s_′ between 5–26 cm^−1^ and μ_a_ between 0–10 cm^−1^. These ranges of optical properties are sufficient to acquire accurate sDRS data for many tissue types between 500–750 nm. Furthermore, our calibration and validation methods were optimized until all average percent errors were below 10% (Figs 6 and 7), a benchmark error value comparable to many similar studies^34^,^35^,^36^,^37^,^39^,^40^,^42^,^43^,^44^,^45^,^47^,^49^,^50^,^51^.

In the same set of calibration phantoms (Fig. 2), sampling depth was determined for each SDS^59^. A demonstration of calculating sampling depth was presented (Fig. 3) and an empirical relationship was determined for sampling depth as a function of μ_a_ and μ_s_′ (Fig. 8). Sampling depths were comparable to a similar study by Hennessy *et al.*^59^.

Once the reflectance LUTs (Fig. 6) and sampling depth LUTs (Fig. 8) were validated, a semi-infinite phantom model was used to simulate dysplastic progression in the oral mucosa (Fig. 4)^1^,^2^,^3^. Results confirmed that the shorter 374 μm SDS was more sensitive to the scattering heterogeneity at superficial layers (Fig. 9), where epithelial dysplasia is known to have a profound effect on the scattering properties in such layers^64^,^65^,^66^. These experiments demonstrate the potential for monitoring scattering changes associated with early epithelial dysplasia which is often confined above the basement membrane^1^,^2^,^3^,^4^.

Next, the bench-top technique was applied to *in vivo* oral mucosa by collecting sDRS data from the inner lip of 13 healthy volunteers (Fig. 5). The LUT-based inverse model was used to extract the wavelength-dependent B, [Hb], and SaO_2_ values from all 13 volunteers (Fig. 10). The representative reflectance data demonstrates increased reflectance for the 730 μm SDS compared to the 374 μm SDS at wavelengths greater than approximately 600 nm, consistent with previous findings^29^,^74^. It is well known that longer SDSs penetrate deeper into tissue, and thus longer wavelengths will dominate reflectance for longer SDSs^51^,^59^,^74^. This phenomenon is apparent when analyzing the scattering exponent (B). At longer separations, B values decrease because of greater reflectance from longer wavelengths.

The extracted absorption-based optical properties, [Hb] and SaO_2,_ were comparable to other studies^35^,^75^. The longer 730 μm SDS extracted greater [Hb] compared to the shorter 374 μm SDS. This supports our hypothesis that the longer SDS sampled deeper into the tissue vasculature, although it is clear the vasculature is still being sampled with the 374 μm SDS^51^,^69^,^72^. This penetration into the vasculature was expected since sampling depth in the short SDS was greater than 300 μm, which exceeds the non-vascularized epithelial thickness of the oral cavity^67^. We anticipate the standard deviations for [Hb] and SaO_2_ values (Fig. 10 and Table 2) to be most likely due to variations in the pressure applied between the probe tip and volunteer’s inner lip. It has been shown that probe-pressure variations among measurements can induce large errors in [Hb] and SaO_2_, so future studies will seek to develop a real-time probe-pressure monitoring system similar in concept to those reported in other studies^76^.

The study presented here was an extensive validation of the quantitative spectroscopy modality of this technique. Since this technique has been validated, its ability to monitor tissue health in response to anti-tumor therapy can be further evaluated in pre-clinical and clinical studies. Additionally, future studies will explore quantitative measures regarding the high-resolution fluorescence imaging modality, such as automated nuclear-to-cytoplasmic ratio and cells-per-area calculations, and co-register these values with sDRS extracted optical parameters. Finally, since this hybrid imaging and spectroscopy technique lacks a widefield imaging modality, future trials will explore designing probes with identical probe-tip geometries that are compatible with conventional endoscopes.

## Conclusion

We have developed a hybrid spectroscopy and imaging technique comprising of a conventional fluorescence fiber-bundle microendoscopy platform coupled with a series of off-axis broadband spectroscopy (sDRS) channels. Since dysplasia can initially arise near the epithelial basement membrane, collecting structural and functional information from deeper within the tissue microenvironment is critical for many applications, including detection of early dysplasia, analysis of tumorigenesis, and monitoring of therapeutic response. As a result, this hybrid imaging and spectroscopy platform may be capable of collecting a wealth of information about the structural and functional properties of tissue at various imaging sites in *ex vivo* and *in vivo* models. Finally, the potential of this technique to be coupled to the biopsy port of a conventional endoscope makes further clinical translation and complimentary optical biopsy in the oral cavity and other epithelial tissues feasible.

## Additional Information

**How to cite this article**: Greening, G. J. *et al.* Towards monitoring dysplastic progression in the oral cavity using a hybrid fiber-bundle imaging and spectroscopy probe. *Sci. Rep.* **6**, 26734; doi: 10.1038/srep26734 (2016).

## Figures and Tables

**Table 1:** Paired t-test statistics for percent increases in μ_s_′ (λ = 630 nm) for dysplasia-mimicking phantom model.

Phantom Comparison	374 μm SDS (n = 10)		730 μm SDS (n = 10)		P-Value	Significance (Y/N), α = 0.01
	Mean	Std. Dev.	Mean	Std. Dev.		
P1 to P2 (%)	4.97	0.40	1.42	1.93	1.67 × 10^−4^	Y
P2 to P3 (%)	16.18	5.95	9.19	1.54	4.58 × 10^−3^	Y
P1 to P3 (%)	21.96	6.42	10.72	0.93	1.23 × 10^−4^	Y

**Table 2:** Paired t-test statistics for extracted *in vivo* oral optical properties from LUT-based inverse model.

Optical Property	374 μm SDS (n = 13)		730 μm SDS (n = 13)		P-Value	Significance (Y/N), α = 0.01
	Mean	Std. Dev.	Mean	Std. Dev.		
B	0.80	0.19	0.48	0.25	8.8 × 10^−4^	Y
[Hb] (mg/mL)	2.39	0.44	2.91	0.65	8.8 × 10^−3^	Y
SaO_2_ (%)	94.1	10.0	91.7	9.10	4.6 × 10^−1^	N
